# High frequency of acquired virulence factors in carbapenemase-producing *Klebsiella pneumoniae* isolates from a large German university hospital, 2013–2021

**DOI:** 10.1128/aac.00602-24

**Published:** 2024-10-04

**Authors:** Janko Sattler, Christoph M. Ernst, Janine Zweigner, Axel Hamprecht

**Affiliations:** 1 Institute for Medical Microbiology, Immunology and Hygiene, University Hospital Cologne and Faculty of Medicine, University of Cologne, Cologne, Germany; 2 Institute of Medical Microbiology and Virology, University of Oldenburg, Oldenburg, Germany; 3 German Center for Infection Research (DZIF), Cologne, Germany; 4 Department of Infection Control and Hospital Hygiene, University Hospital Cologne and Faculty of Medicine, University of Cologne, Cologne, Germany; University of Fribourg, Fribourg, Switzerland

**Keywords:** *Klebsiella pneumoniae*, virulence factors, hypervirulent *Klebsiella pneumoniae*, convergence, multidrug resistance, carbapenem resistance, surveillance, carbapenemase, aerobactin

## Abstract

Carbapenemase-producing *Klebsiella pneumoniae* (CP-Kp) isolates are a public health concern as they can cause severe hospital-acquired infections that are difficult to treat. It has recently been shown that CP-Kp can take up virulence factors from hypervirulent *K. pneumoniae* lineages. In this study, 109 clinical CP-Kp isolates from the University Hospital Cologne were examined for the presence of acquired virulence factors using whole-genome sequencing and phenotypic tests, and results were linked to clinical data. The virulence factor *iuc* was present in 18/109 of the CP-Kp isolates. Other acquired virulence factors, such as *ybt*, *cbt*, *iro*, *rmpA/rmpA2*, *peg-344*, and hypervirulence-associated capsule types were detected in various combinations among these isolates. The *iuc*-positive isolates produced OXA-232 (*n* = 7), OXA-48 (*n* = 6), OXA-48+NDM (*n* = 3), NDM, and KPC (each *n* = 1), and 7/18 isolates were resistant to ceftazidime-avibactam, colistin, and/or cefiderocol. Four isolates carried hybrid plasmids that harbored acquired virulence factors alongside the carbapenemase genes *bla*_NDM-1/5_ or *bla*_OXA-48_. In 15/18 patients, *iuc*-positive CP-Kp were isolated from a clinically manifest infection site. Among these, four patients had osteomyelitis, and four patients died from pneumonia with OXA-232-producing ST231 isolates, three of them as part of an outbreak. In conclusion, acquired virulence factors are frequently detected in various combinations in carbapenemase-producing *K. pneumoniae* isolates in Germany, warranting continuous monitoring of infections caused by these strains.

## INTRODUCTION

*Klebsiella pneumoniae* is a clinically relevant pathogen that is responsible for a large portion of hospital-acquired infections. In Europe, most of the clinical *K. pneumoniae* isolates belong to the classical *K. pneumoniae* group (cKp) (1). Classical *K. pneumoniae* are opportunistic pathogens that cause severe infections mainly in medically compromised patients, for example, hemato-oncology patients or patients in intensive care units. However, these strains have a high propensity for the acquisition of resistance genes, which can severely limit the treatment options. The standard therapy for severe infections with Gram-negative bacteria often comprises carbapenem antibiotics, due to their broad range, high bactericidal activity, and few adverse effects. However, a Europe-wide analysis of the European Centre for Disease Prevention and Control (ECDC) has found that the carbapenem resistance rate in invasive *K. pneumoniae* isolates is high in many countries and even exceeds 50% in Greece and Russia (2). Carbapenem resistance in *K. pneumoniae* is most often caused by the production of carbapenemases; enzymes, that inactivate carbapenems and most other β-lactam antibiotics. Moreover, carbapenemase-producing *K. pneumoniae* (CP-Kp) can also be resistant to last-line antibiotics such as cefiderocol (3), ceftazidime-avibactam, and even aztreonam-avibactam (4).

Besides cKp, there is another group of *K. pneumoniae*, called hypervirulent *K. pneumoniae* (hvKp). As opposed to cKp, these strains can cause severe community-acquired infections in otherwise healthy young individuals, which can manifest in unusual infection sites, including hepatic abscesses, endophthalmitis, osteomyelitis, etc. (5). Their hypervirulent phenotype is linked to certain capsule types, such as K1, K2, and K20, as well as the presence of virulence factors that are usually situated on large hypervirulence plasmids, like pK2044 (6). These virulence factors comprise the siderophores aerobactin (*iuc*), yersiniabactin (*ybt*), salmochelin (*iro*), the genotoxin colibactin (*cbd*), the *rmpA/rmpA2* locus, responsible for the hypermucous phenotype, and *peg-344*, a putative metabolite transporter (7). Among these, *iuc* is probably the major virulence factor of hvKp (8).

Conventionally, hvKp and cKp lineages are assigned to distinct clonal groups, such as GC23 for hvKp and GC258 for cKp. However, recent reports have shown that cKp can also acquire virulence factors through the uptake of hypervirulence plasmid parts (9), which has led to the fear of a convergence of carbapenem resistance and hypervirulence in *K. pneumoniae* (10). Until now, the clinical significance of acquired virulence factors in cKp is not clear. While recent studies suggest that the acquisition of virulence factors from hvKp does not lead to a hypervirulence phenotype in mice in most cases (11), the potential for a more gradual impact on virulence or colonization remains unclear because of the general difficulty of establishing infections in mice with cKp.

Classical CP-Kp isolates with acquired virulence factors have primarily been described in Asia (12), but some reports also indicate their presence in Europe (13, 14). For Germany, the prevalence and genomic background of these isolates are unclear, as reports have been limited to outbreaks (15).

In this study, we examined the presence of acquired virulence factors in a large collection of CP-Kp isolates from Germany and linked the molecular information with the associated clinical data.

## MATERIALS AND METHODS

### Isolates and antibiotic susceptibility testing

A total of 109 non-redundant CP-Kp isolates were collected between 2013 and 2021 from clinical samples at the University Hospital Cologne. Identification was carried out with MALDI-TOF MS (Bruker Daltonic, Bremen, Germany), and the presence of carbapenemase genes was confirmed by PCR (GeneXpert Carba-R assay, Cepheid, Frankfurt, Germany) as previously described (16). Minimal inhibitory concentrations (MICs) for antibiotics were determined by broth microdilution (BMD) employing the Micronaut-S GN3 panel (Merlin, Bornheim, Germany) except for cefiderocol, which was tested using the UMIC Cefiderocol BMD strip (Bruker Daltonics, Bremen, Germany). The results were interpreted according to EUCAST clinical breakpoints (version 14.0).

### Sequencing and bioinformatic analysis

All CP-Kp isolates underwent whole-genome sequencing (WGS) with short-read technique (Illumina, San Diego, CA, United States) as previously described (17). Isolates carrying the virulence factor *iuc*, further referred to as CP-Kp/*iuc*, underwent long-read sequencing (Oxford Nanopore Technologies, Oxford, UK), and hybrid assemblies were created with unicycler (18). Assemblies were analyzed for the presence of acquired virulence factors, as well as the species subtype and MLST type with Kleborate (19). Comparative genomic analyses were performed with BLAST+ (20). To determine highly similar plasmids, a cut-off was set to ≥90% query coverage and ≥99% similarity. Whole-genome single nucleotide polymorphism (SNP) phylogeny analysis was performed with CSIPhylogeny (21).

### Phenotypic hypermucoidity assays

The string test was performed using Columbia blood agar as described before (22). Centrifugation resistance, as a quantitative indicator of hypermucoidity, was performed as described before, measuring the optical density at 600 nm (OD_600_) after centrifugation (23). The hvKp isolate SGH10 (24) was used as a positive control. As OD_600_ before centrifugation was normalized, the result was given as absolute OD_600_ after centrifugation. For each sample, OD_600_ was measured from separate cultures on two different days in triplicates, and the mean and standard deviation of the six obtained values was reported.

## RESULTS

### Characterization of aerobactin positive isolates

Within the collection of 109 CP-Kp, the aerobactin gene *iuc* was present in 18 isolates (16.5%). These isolates were selected for further analysis (Table 1). The CP-Kp/*iuc* strains were isolated at high rates from putative infection sites (as opposed to screening material) in 15/18 patients (83%), compared to 52/91 (57%) in *iuc*-negative CP-Kp. Furthermore, the specimen type indicated high invasiveness of the CP-Kp/*iuc* isolates (i.e., blood culture, bone) in 9/18 patients (50%), compared to 1/91 (1%) in *iuc*-negative CP-Kp. The most frequently associated infection with CP-Kp/*iuc* strains was pneumonia (*n* = 7), followed by osteomyelitis (*n* = 4) (Table 1). In four cases, pneumonia with the isolated CP-Kp was considered responsible for the fatal outcome of the patient, as assessed by the treating clinician. In two cases, chronic osteomyelitis was refractory to repeated surgical debridement leading to the medical indication of limp amputation. With the exception of the CP-Kp-ST231 outbreak cluster (see paragraph “Outbreak of CP-Kp-ST231/*iuc5*”), most patients had a non-German background, with the highest numbers being from Russia (*n* = 5) and Libya (*n* = 3).

**TABLE 1:** Isolate and patient metadata of *iuc*-positive carbapenemase-producing *K. pneumoniae* isolates from the University Hospital Cologne, Germany, 2013–2021

Isolate	Isolation year	ST type	Carbapenemase	Material^*a*^	Infection focus	Fatal outcome	Origin of patient
KLPN-IUC-01	2013	11	OXA-48	Wound swab	Wound infection	NA	NA
KLPN-IUC-02	2014	395	KPC-3	Stool	Colonization	Yes (NI)	Germany
KLPN-IUC-03	2016	395	OXA-48	Bone	Osteomyelitis	No	Russia
KLPN-IUC-04	2016	147	NDM-1	Rectal swab	Colonization	No	Russia
KLPN-IUC-05	2019	231	OXA-232	Blood culture, respiratory	Pneumonia	Yes (NI)	Sudan
KLPN-IUC-06	2019	231	OXA-232	Respiratory, urine	Pneumonia	Yes	Germany
KLPN-IUC-07	2019	231	OXA-232	Blood culture, respiratory	Pneumonia	Yes	Germany
KLPN-IUC-08	2019	231	OXA-232	Blood culture, respiratory	Pneumonia	No	Germany
KLPN-IUC-09	2019	231	OXA-232	Respiratory	Pneumonia	Yes	Germany
KLPN-IUC-10	2019	395	OXA-48	Rectal swab	Colonization	No	Russia
KLPN-IUC-11	2019	231	OXA-232	Urine	Urinary tract infection	No	Germany
KLPN-IUC-12	2020	395	OXA-48	Throat swab, blood culture	Pneumonia	No	Russia
KLPN-IUC-13	2020	147	OXA-48, NDM-1	Bone	Osteomyelitis	No	Libya
KLPN-IUC-14	2020	268	OXA-48	Urine	Urinary tract infection	No	Germany
KLPN-IUC-15	2021	383	OXA-48, NDM-5	Bone	Osteomyelitis	No	Libya
KLPN-IUC-16	2021	15	OXA-48, NDM-1	Bone	Osteomyelitis	No	Libya
KLPN-IUC-17	2021	395	OXA-48	Blood culture	Neutropenic colitis	No	Russia
KLPN-IUC-18	2021	231	OXA-232	Respiratory	Pneumonia	Yes	Iraq

^*a*^ Material contains only the most clinically relevant specimens from which the bacterium was isolated. NA = not available, NI = not infection-related.

**FIG 1:**
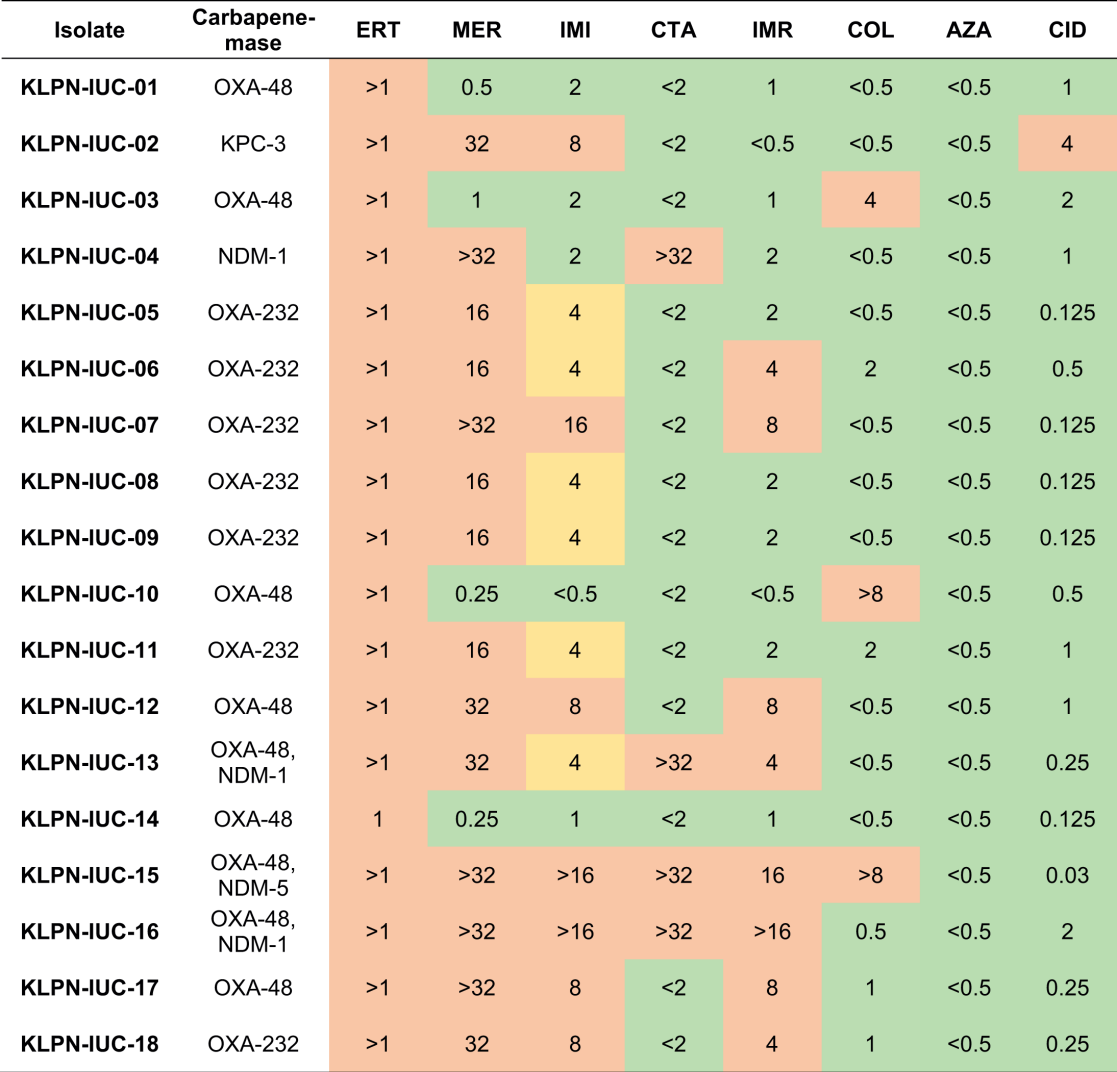
Minimal inhibitory concentrations (MICs) for last-resort antibiotics of *iuc*-positive carbapenemase-producing *K. pneumoniae* isolates from clinical samples at the University Hospital Cologne, Germany, 2013–2021. Interpretation of MICs according to EUCAST breakpoints is colour encoded; red = resistant; yellow = susceptible, increased exposure; green = susceptible, standard dosing regimen. For aztreonam-avibactam, breakpoints for aztreonam were applied. AZA, aztreonam-avibactam; CID, cefiderocol; COL, colistin; CTA, ceftazidime-avibactam; ERT, ertapenem; IMI, imipenem; IMR, imipenem-relebactam; MER, meropenem.

Antibiotic susceptibility testing for last resort antibiotic showed that 4/18 isolates (22%), all NDM producers, were resistant to ceftazidime-avibactam as expected, 3/18 isolates (17%) were resistant to colistin, and 1/18 isolates (6%) was resistant to cefiderocol (Fig. 1).

### Genomic analysis

**FIG 2:**
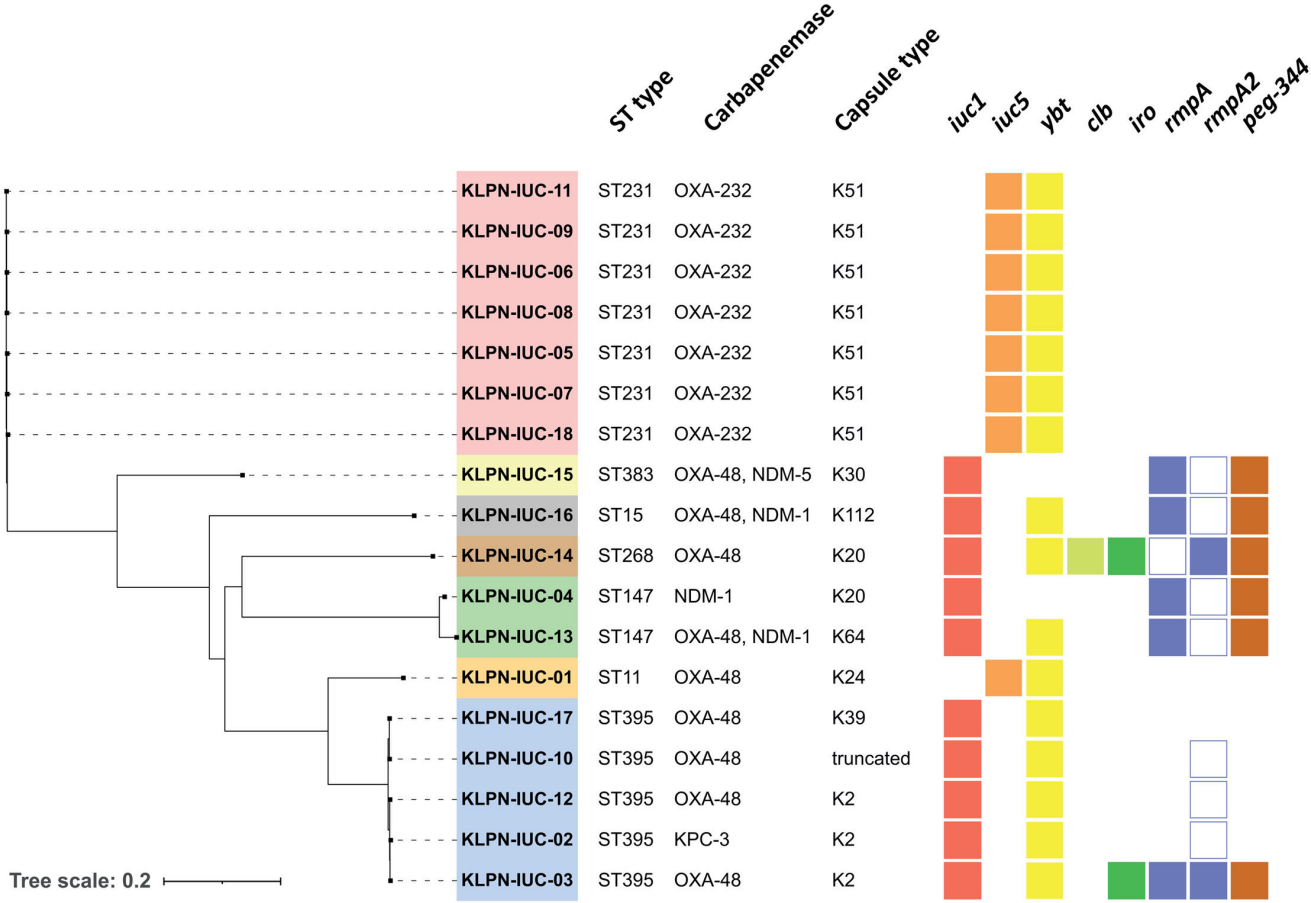
Whole-genome SNP phylogeny, sequence type (ST), presence of carbapenemase genes and genetic hypervirulence markers of *iuc*-positive carbapenemase-producing *K. pneumoniae* isolates from clinical samples at the University Hospital Cologne, Germany, 2013–2021. Isolate names are colour-encoded according to their ST type. The presence of a complete hypervirulence gene is indicated by a filled box. The presence of a truncated hypervirulence gene is indicated by an empty box. The tree scale indicates SNPs per aligned genome (80% of the reference genome) in per cent.

Analysis of the ST type revealed that none of the 18 *iuc*-positive isolates belonged to a hypervirulence-associated clonal lineage, but all had a cKp background. Apart from the outbreak strain CP-Kp-ST231, the most frequent ST types were ST395 (*n* = 5) and ST147 (*n* = 2) (Fig. 2 and Table 1). Different aerobactin gene types were detected, as classified by Lam *et al*. (25). While most ST types carried *iuc1*, which is associated with the typical hypervirulence plasmids, isolates with ST11- and ST231-harbored *iuc5* on a 71 kbp-sized IncFIA plasmid, and no sequence parts of a hypervirulence plasmid were present in these isolates. In all *iuc1*-positive isolates, other acquired virulence factors (*rmpA/A2*, *iro, clb, peg-344*) were found in different combinations, depending on which regions of the hypervirulence plasmid were present in the isolate (Fig. 2). The yersiniabactin gene *ybt* was detected in 17/19 isolates and was embedded within variants of ICE*Kp* on the chromosome. Five isolates carried capsule types associated with hypervirulence, which were K2 and K20. Two isolates, KLPN-IUC-03 and −14, fulfilled genomic criteria for hypervirulence in a stricter sense, harboring *iuc1, iro*, *rmpA*, *rmpA2*, *peg-344,* and a K2 or K20 capsule type.

Among all CP-Kp/*iuc* isolates, *bla*_OXA-48_ was the most prevalent carbapenemase gene (*n* = 9), and three of these isolates co-harbored *bla*_NDM-1_ or *bla*_NDM-5_. The outbreak strain CP-Kp-ST231/*iuc5* and an ST11 isolate harbored *bla*_OXA-232_ (*n* = 6), and two isolates harbored either *bla*_KPC-3_ or *bla*_NDM-1_ alone, respectively.

### Hypermucoidity assays

Only one CP-Kp/*iuc* isolate, KLPN-IUC-03, was positive for the string test (Table 2). This isolate carried intact *rmpA* and *rmpA2* genes and a K2 capsule type. Only one isolate, KLPN-IUC-02, showed a high turbidity in the centrifugation resistance test, with an OD_600_ of 1.03 (reference value of hvKp SGH10 = 1.35). Interestingly, this isolate did not carry an *rmpA* gene, and *rmpA2* was truncated (Kleborate match: rmpA2_6*−50%), but it had a K2 capsule and carried a nonsynonymous point mutation in the Walker A’ Box of the capsule biosynthesis enzyme Wzc, involved in capsule polymerization. All other isolates were either negative (OD_600_ <0.1) (*n* = 12), or they displayed a low-level centrifugation resistance (OD_600_ 0.10–0.43) (*n* = 5) (Table 2).

**TABLE 2:** Results of phenotypic assays for the detection of hypervirulence in *iuc*-positive carbapenemase-producing *K. pneumoniae* isolates from the University Hospital Cologne, Germany, 2013–2021^*a*^

Isolate	String test	Centrifugation resistance OD_600_
KLPN-IUC-01	Negative	0.03 (SD 0.006)
KLPN-IUC-02	Negative	1.03 (SD 0.058)
KLPN-IUC-03	Positive	0.43 (SD 0.133)
KLPN-IUC-04	Negative	0.03 (SD 0.006)
KLPN-IUC-05	Negative	0.04 (SD 0.014)
KLPN-IUC-06	Negative	0.05 (SD 0.009)
KLPN-IUC-07	Negative	0.05 (SD 0.009)
KLPN-IUC-08	Negative	0.04 (SD 0.014)
KLPN-IUC-09	Negative	0.06 (SD 0.024)
KLPN-IUC-10	Negative	0.02 (SD 0.005)
KLPN-IUC-11	Negative	0.05 (SD 0.007)
KLPN-IUC-12	Negative	0.12 (SD 0.045)
KLPN-IUC-13	Negative	0.1 (SD 0.055)
KLPN-IUC-14	Negative	0.04 (SD 0.016)
KLPN-IUC-15	Negative	0.04 (SD 0.019)
KLPN-IUC-16	Negative	0.22 (SD 0.083)
KLPN-IUC-17	Negative	0.17 (SD 0.021)
KLPN-IUC-18	Negative	0.05 (SD 0.02)

^*a*^ SD = standard deviation.

### Outbreak of CP-Kp-ST231/*iuc5*

Detection of CP-Kp-ST231/*iuc5* was associated with a hospital outbreak in six patients. Phylogenetic analysis showed that the isolates KLPN-IUC-05, -06, -07, -08, -09, and -11 formed a closely related cluster with a maximum of seven SNPs distance (Fig. S1). In comparison, external isolates of the same ST type and KLPN-IUC-18 were on average 72–138 SNPs different from the cluster. The index patient was already colonized on admission and stayed in the hospital for 8 months. During that time, CP-Kp-ST231/*iuc5* was isolated in five other patients. All infected patients had overlapping ward stays with other patients from the cluster and the transmission chains were confirmed by investigations of the infection control department. Five of the six outbreak patients with CP-Kp-ST231/*iuc5* had pneumonia; three of these died from septic complications during their intensive care unit stay.

### Carbapenemase/hypervirulence hybrid plasmids

**FIG 3:**
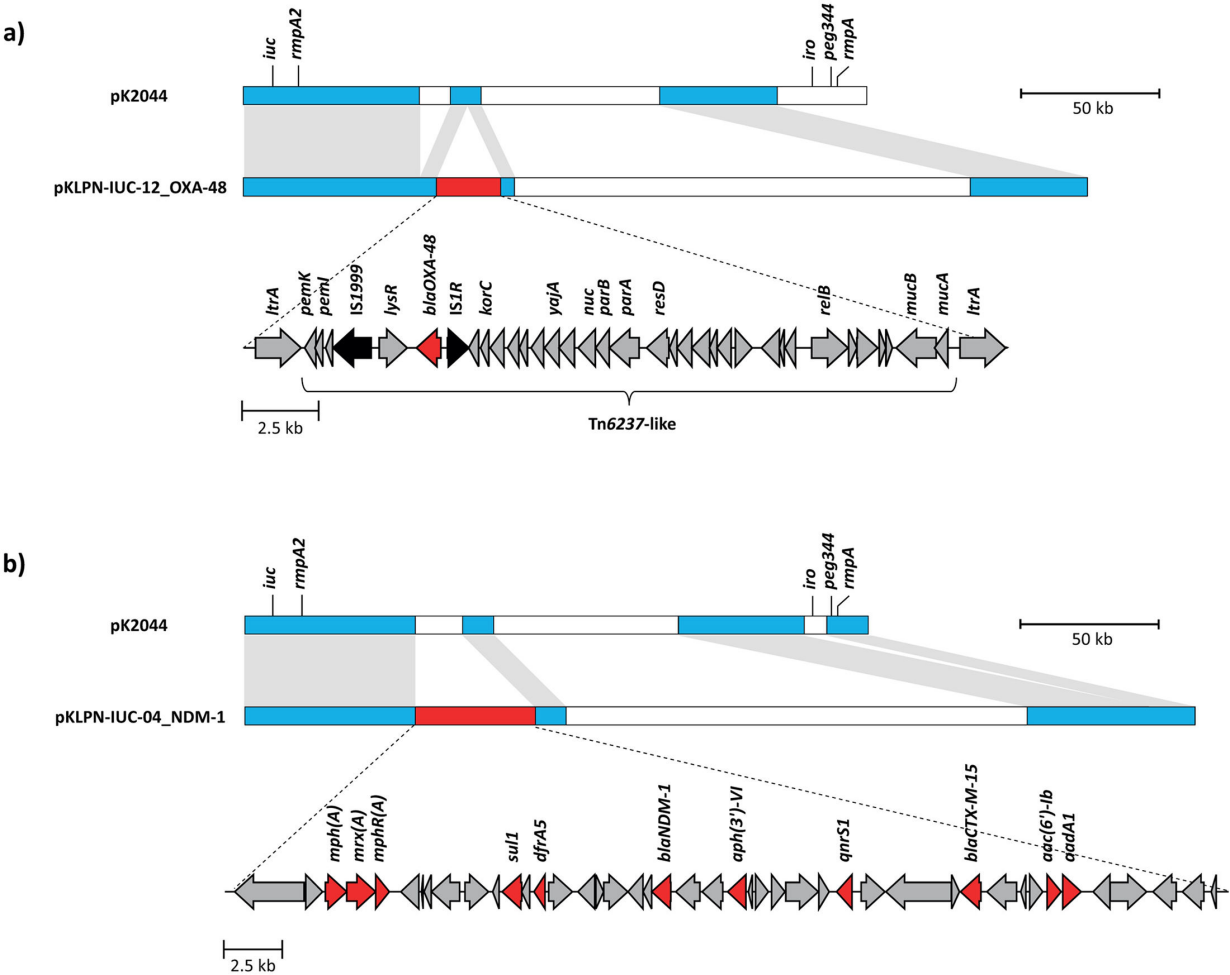
Genetic composition of the carbapenemase/hypervirulence hybrid plasmids pKLPN-IUC-12_OXA-48 (a) and pKLPN-IUC-NDM-1 (b). Blue regions show strong sequence homology between the hypervirulence plasmid pK2044 and the hybrid plasmids. Red regions carry the carbapenemase genes. White regions are neither related to a hypervirulence nor a carbapenemase gene environment. Red arrows = genes associated with antibiotic resistance, black arrows = insertion sequences, grey arrows = other genes or open reading frames.

In four isolates, acquired virulence factors and carbapenemase genes were located on the same hybrid plasmids. KLPN-IUC-12 harbored *bla*_OXA-48_ on a 305 kbp IncHI1B plasmid (pKLPN-IUC-12_OXA-48), whereby 140 kb of the plasmid showed strong sequence homology with the hypervirulence plasmid pK2044 (Fig. 3a). In pKLPN-IUC-12_OXA-48, *bla*_OXA-48_ was integrated within a variant of Tn*6237*, here referred to as Tn*6237*-like, flanked by two identical copies of *ltrA*. In Tn*6237-like*, invTn*1999* is located downstream of *korC* together with ∆*tir*, *pemI,* and *pemK* compared to Tn*6237*. The hybrid plasmid in KLPN-IUC-10 is highly similar to pKLPN-IUC-12_OXA-48, but some sequence parts belonging to the pK2044 plasmid have been truncated, resulting in a smaller size of 283 kbp. However, this did not affect the regions with known virulence factors.

KLPN-IUC-04 harbored *bla*_NDM-1_ on a 342 kbp plasmid with an IncFIB and IncHI1B determinant. This plasmid, pKLPN-IUC-04-NDM-1, showed strong sequence homology with pK2044 in a region of 134 kbp (Fig. 3b). Within the plasmid, *bla*_NDM-1_ was integrated into a 43 kbp antimicrobial resistance region, carrying antibiotic resistance genes against β-lactam antibiotics, fluoroquinolones, aminoglycosides, macrolides, and trimethoprim as well as genes associated with heavy metal resistance. The 344 kbp-sized plasmid of KLPN-IUC-15 is largely identical to pKLPN-IUC-04-NDM-1, including the replicon types, but it shows some rearrangements of larger regions. Furthermore, it carries *bla*_NDM-5_ instead of *bla*_NDM-1_.

To estimate whether these hybrid plasmids are endemic in Europe, their sequences were aligned with publicly available sequences on GenBank using BLAST+. As shown in Table 3, the two plasmid types showed most high-similarity matches with isolates from Russia (*n* = 6 and 7), but the *bla*_NDM_-carrying plasmid also showed four matches with isolates from Italy and two with Great Britain and Poland each.

**TABLE 3:** Number of high similarity GenBank BLAST matches (≥90% query coverage and ≥99% similarity) for hybrid plasmids pKLPN-IUC-12_OXA-48 and pKLPN-IUC-04_NDM-1 and country of submission^*a*^

	RUS	USA	CHN	CHE	EGY	ITA	GBR	CZE	POL	QAT
pKLPN-IUC-12_OXA-48	6	2	1	1	0	0	0	0	0	0
pKLPN-IUC-04_NDM-1	7	6	1	0	1	4	2	1	2	1

^*a*^ As the results for both *bla*_OXA-48_- and *bla*_NDM_-carrying hybrid plasmids were identical, only one result per carbapenemase type is shown. CHE = Switzerland, CHN = China, CZE = Czechia, EGY = Egypt, GBR = United Kingdom, ITA = Italy, POL = Poland, QAT = Qatar, RUS = Russian Federation, USA = United States.

## DISCUSSION

This study discovered a high rate of acquired virulence factors in clinical CP-Kp isolates. With 16.5% of the isolates being *iuc*-positive, this rate is higher than previously reported values, with 5.6% in a study of an older CP-Kp collection in Germany (26) and 9.0% in a similar study on Swiss isolates (14). This higher share is potentially due to the large number of international patients from high-endemicity areas at our institution and the inclusion of several isolates from an outbreak.

The clinical significance of acquired virulence factors in cKp isolates needs to be assessed carefully. On one hand, these isolates usually do not show typical clinical manifestations of hvKp strains, that is, liver abscesses, endophthalmitis etc. On the other hand, such isolates are regularly reported in the context of fatal clinical outbreaks (9). In a recent study, where hvKp strains were defined by a median lethal dose (LD_50_) of less than 10^7^ colony-forming units in a mouse model of infection, the acquisition of multiple virulence factors in strains with cKp associated capsule types did not lead to a hvKp phenotype in mice (11). This is in line with the clinical data from our study, which indicates that the acquisition of virulence factors by cKp does not promote hypervirulence in the form of systemic infection, as seen in hvKp isolates. However, due to the well-established function of most of these virulence factors (8), it is conceivable that these isolates are capable of complicating infections in hospitalized patients, for example, by increased persistence or incremental increase of invasiveness. A potential impact on infection persistence would be in line with the four cases of osteomyelitis, a rather uncommon site for cKp infections. Of these, two (50%) were refractory to repeated surgical debridement in combination with antibiotic therapy. Furthermore, CP-Kp/*iuc* isolates were isolated at a higher ratio from invasive material compared to *iuc*-negative CP-Kp isolates. In light of this and recent studies, it is evident that better animal models and more detailed clinical data are necessary to further assess the association between certain acquired virulence factors and clinical outcomes outside of hvKp lineages.

The convergence of virulence factors and multidrug resistance determinants on hybrid plasmids is concerning, as this facilitates the combined spread of these factors in an extremely vulnerable patient population that is already dealing with multidrug-resistant cKp. Hybrid plasmids carrying carbapenemase genes, mainly *bla*_NDM_, were first described in 2019 (27) and have since regularly been reported, for example, in Russia (28). All hybrid plasmids detected in our study are from patients with a non-German background, and, based on GenBank BLAST analysis, these plasmids do not seem to be endemic in Europe at the moment. Nevertheless, *bla*_NDM_-carrying hybrid plasmids were detected sporadically in several countries in Europe (Table 3), and recently, an outbreak of ST147 with a *bla*_NDM-1_-carrying hybrid plasmid highly similar to KLPN-IUC-04 has been reported in Italy (29), indicating that the spread of such plasmids in Europe might be impending.

In general, CP-Kp/*iuc* isolates from our study were mostly recovered from patients with a non-German background, mainly Russia and Libya. This confirms the importance of multidrug resistance screening for patients from endemic areas or other high-risk backgrounds. And, while this study only includes isolates until 2021, the risk of importing CP-Kp/*iuc* to Germany has even increased by now through refugees from Ukraine, as highlighted recently (30). Therefore, our data warrant continuous surveillance monitoring for infections caused by potentially increasingly virulent, extremely antibiotic-resistant strains.

The strength of this study lies in the large isolate collection that comprises clinical CP-Kp over the time span of 9 years. The availability of short- and long-read WGS data allowed for the thorough analysis of genetic content and structural elements like hybrid plasmids, and the access to clinical data for almost all isolates allowed for a clinical association of these data. The main limitation of the study is that it was designed to focus on genomic virulence factors for the isolates, without including animal models of virulence or persistence that may be able to detect gradual increases of pathogenicity in cKp. Another limitation is that the study was conducted as a single-centre study and might therefore not be representative of the general epidemiology of CP-Kp/*iuc* in Germany.

In conclusion, this study showed that acquired virulence factors were frequently detected in various combinations in a collection of carbapenemase-producing *K. pneumoniae* at a German university hospital, indicating that they are becoming prevalent. This study advocates the molecular surveillance of virulence factors in *K. pneumoniae*, which should particularly cover patients from high-endemicity areas.

## Data Availability

The complete nucleotide sequence assemblies of all CP-Kp/*iuc* isolates were deposited at DDBJ/ENA/GenBank under BioProject no. PRJNA1060526.

## SUPPLEMENTAL MATERIAL

Figure S1 aac.00602-24-s0001.docx Whole-genome phylogeny of CP-Kp-ST231/*iuc5* isolates.

ASM does not own the copyrights to Supplemental Material that may be linked to, or accessed through, an article. The authors have granted ASM a non-exclusive, world-wide license to publish the Supplemental Material files. Please contact the corresponding author directly for reuse.
