# Supplementary material for: High frequency of acquired virulence factors in carbapenemase-producing Klebsiella pneumoniae isolates from a large German university hospital, 2013–2021
Source: Antimicrob Agents Chemother. 2024 Oct 4;68(11):e00602-24. doi: 10.1128/aac.00602-24 (PMC11539210; doi:10.1128/aac.00602-24)
Supplement: Figure S1 — Whole-genome phylogeny of CP-Kp-ST231/iuc5 isolates. [file aac.00602-24-s0001.docx]

**High frequency of hypervirulence markers in carbapenemase-producing *Klebsiella pneumoniae* isolates from a large German university hospital, 2013 to 2021**

Janko Sattler^1,2,3,*^, Christoph M. Ernst^1,3^, Janine Zweigner^4^, Axel Hamprecht^2,3^

**Supplementary material**

**
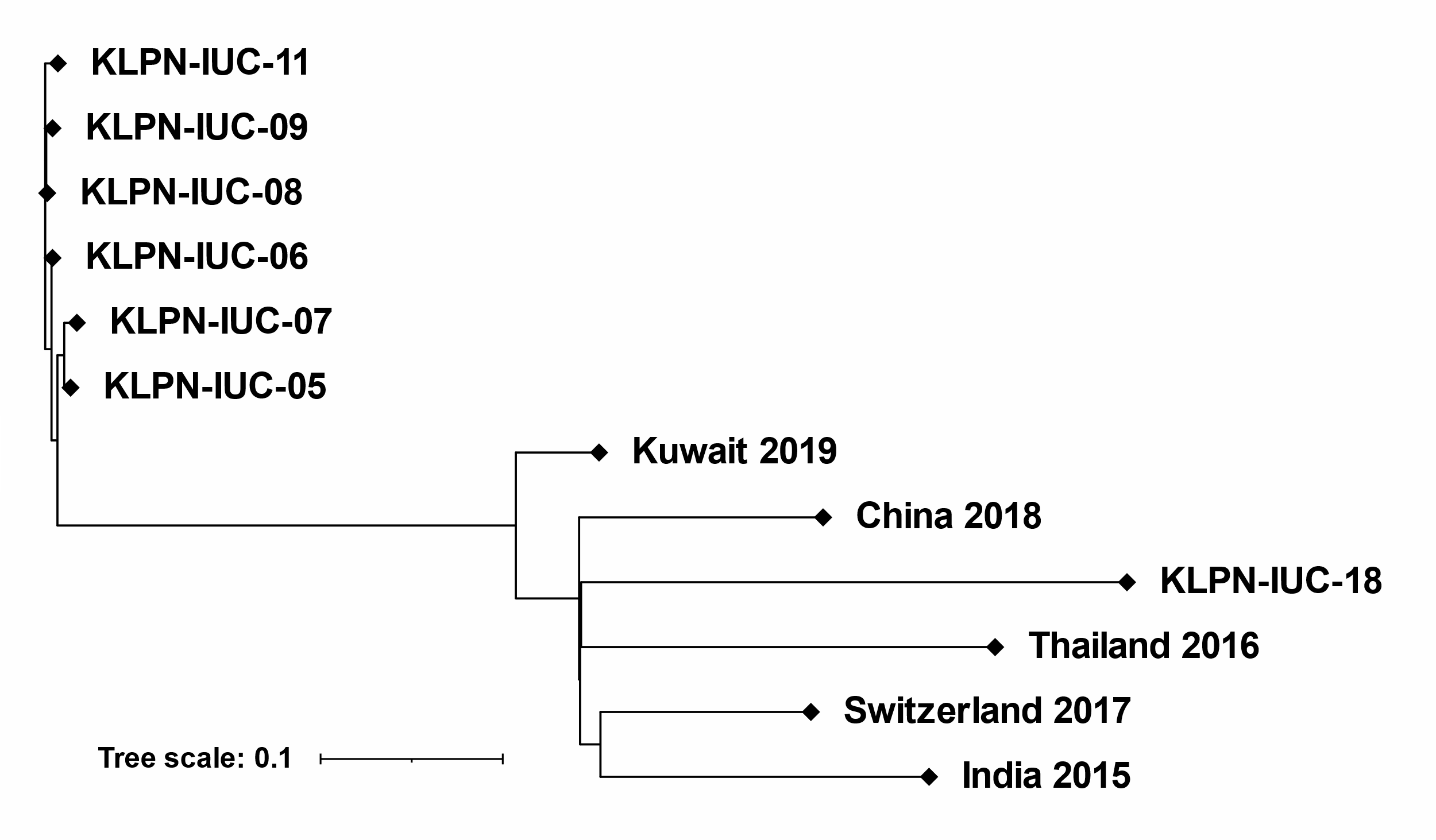
**

**Supplementary Figure 1** Whole genome phylogeny of CP-Kp-ST231/*iuc5* isolates collected at the University Hospital Cologne, Germany, 2019 to 2021, and selected publicly available external strain sequences, indicated by the country and year of isolation.

GenBank or Refseq accession numbers are as follows: Kuwait DAGKLZ000000000, China GCF_024357615, Thailand GCF_002251895, Switzerland NTFP00000000, India GCF_001758405. The tree scale indicates SNPs per aligned genome (92% of the reference) in per cent.
